# Research and Remedies

**Published:** 1913-06-14

**Authors:** 


### RESEARCH AND REMEDIES.

### Surgery and Life Assurance.

It is the experience of all life assurance companies that nowadays their clients live on the average distinctly longer than the "expectation of life" for their ages, as derived from the statistics of a generation or so ago. This state of things is, of course, of benefit to the insured as well as to those who assure them: the former live longer, the latter make more profit out of their business. The advance of modern surgery must surely be responsible for some part of this prolongation of the average life, though medicine (especially preventive medicine) may claim a larger share still. At any rate, the actuaries and medical officers of assurance companies have to keep constantly in touch with surgical aims and accomplishments, an interesting illustration of which fact is afforded by an article in the New York *Medical Record* concerning the insurability of women who have undergone hysterectomy or ovariotomy. The details of the argument cannot be reproduced here, but the author's conclusions are of some moment and interest. He holds that such women can and ought to be accepted at ordinary rates on four conditions. First, that no malignancy was expected or found at the time of operation. Secondly, that the operation revealed no complication or likelihood of one. Thirdly, that at least two years have elapsed since the operation to make sure that all nervous and physical disturbance has entirely disappeared. Lastly, that the applicant is up to the required standard of health in all other respects.

### Graves's Disease.

In the *Medical Press* Dr. Herbert French lays stress upon the risk of partial thyroidectomy for Graves's disease, and advises various alternatives which are worth trying before any decision to operate is arrived at. Rest in bed and feeding up; *x*-rays locally; and the interrupted galvanic current are some of these measures. Beyond these he speaks very enthusiastically of the application of suprarenal extract (one in a thousand) on lint to the whole front of the neck. As a rule he prescribes this for night only; the lint is covered with gutta-percha tissue to keep it moist, and is applied so wet that it just does not drip. Dr. French gives no reasoned explanation of this method of treatment, nor any statistics as to its effect: he merely records his general impression that clinically the results are good. It would be stupid to belittle the clinical impressions of so acute an observer as Dr. French, who himself admits that he is far from being able to offer any rigid scientific proof of his theorem. But we fancy that Dr. Short, whose book on "Physiology in Surgical Practice" created such a reputation for high thinking a year or two ago, would have some serious objections to urge against their acceptance. Still, we cannot yet afford to throw aside empiricism in medicine; and if further experiment by impartial observers shows that adrenalin locally applied really does help exophthalmic goitre cases, the fact that it is difficult to see why will not count for very much with those who have the actual care of these patients.

